# How yeast re-programmes its transcriptional profile in response to different nutrient impulses

**DOI:** 10.1186/1752-0509-5-148

**Published:** 2011-09-25

**Authors:** Duygu Dikicioglu, Erkan Karabekmez, Bharat Rash, Pınar Pir, Betul Kirdar, Stephen G Oliver

**Affiliations:** 1 Department of Chemical Engineering, Bogazici University, Bebek 34342, Istanbul, Turkey; 2 Faculty of Life Sciences, University of Manchester, M1 9PT, Manchester, UK; 3 Cambridge Systems Biology Centre & Department of Biochemistry, University of Cambridge, CB2 1GA, Cambridge, UK; 4 Novacta Biosystems Limited, BioPark Hertfordshire, Welwyn Garden City, AL7 3AX, Herts, UK

## Abstract

**Background:**

A microorganism is able to adapt to changes in its physicochemical or nutritional environment and this is crucial for its survival. The yeast, *Saccharomyces cerevisiae*, has developed mechanisms to respond to such environmental changes in a rapid and effective manner; such responses may demand a widespread re-programming of gene activity. The dynamics of the re-organization of the cellular activities of *S. cerevisiae *in response to the sudden and transient removal of either carbon or nitrogen limitation has been studied by following both the short- and long-term changes in yeast's transcriptomic profiles.

**Results:**

The study, which spans timescales from seconds to hours, has revealed the hierarchy of metabolic and genetic regulatory switches that allow yeast to adapt to, and recover from, a pulse of a previously limiting nutrient. At the transcriptome level, a glucose impulse evoked significant changes in the expression of genes concerned with glycolysis, carboxylic acid metabolism, oxidative phosphorylation, and nucleic acid and sulphur metabolism. In ammonium-limited cultures, an ammonium impulse resulted in the significant changes in the expression of genes involved in nitrogen metabolism and ion transport. Although both perturbations evoked significant changes in the expression of genes involved in the machinery and process of protein synthesis, the transcriptomic response was delayed and less complex in the case of an ammonium impulse. Analysis of the regulatory events by two different system-level, network-based approaches provided further information about dynamic organization of yeast cells as a response to a nutritional change.

**Conclusions:**

The study provided important information on the temporal organization of transcriptomic organization and underlying regulatory events as a response to both carbon and nitrogen impulse. It has also revealed the importance of a long-term dynamic analysis of the response to the relaxation of a nutritional limitation to understand the molecular basis of the cells' dynamic behaviour.

## Background

The ability of a microorganism to adapt changes in its physicochemical (e.g. temperature [1], pH [2]) or nutritional [3,4] environment is crucial for its survival. The yeast, *Saccharomyces cerevisiae*, has developed mechanisms to respond to such environmental changes in a rapid and effective manner; such responses may demand a widespread re-programming of gene activity [2,5]. Among other transcription factors, Msn2p and Msn4p regulate the expression of ~200 genes in response to several stresses, including heat shock, osmotic shock, oxidative stress, low pH, glucose starvation, sorbic acid and high ethanol concentrations [6]. This is especially true of changes in the nutrient environment and the ability to sense and respond to changes in nutrient availability is essential for cells from both unicellular and multicellular organisms. Glucose is the most abundant monosaccharide on earth and is the preferred carbon source for most organisms and, accordingly, changes in glucose availability often have profound consequences in many types of cell [7]. A negative regulator of the glucose-sensing signal transduction pathway, Mth1p, is required for repression of transcription by Rgt1p. Mth1p interacts with Rgt1p and the glucose sensors Snf3p and Rgt2p to play one of the key regulatory roles in response to the amount of available glucose in the environment [8]. Gcr1p and Gcr2p are transcription factors that activate genes involved in glycolysis, and are also among the major regulators mediating carbon catabolite repression [9]. The introduction of glucose to a culture of *S. cerevisiae *cells growing by respiration evokes changes at both the level of gene expression and of metabolism, with several proteins being activated or deactivated and gene expression being completely re-programmed to accommodate the switch from respiration to fermentation. Many transcriptional regulators are involved in the process including the HAP complex, which is a transcriptional activator and a global regulator of respiratory gene expression [10]. Carbon catabolite repression down-regulates the expression of genes that encode enzymes involved in gluconeogenesis, the Krebs cycle, respiration, mitochondrial development, and the utilization of carbon sources other than glucose, fructose or mannose [11]. While the main effect of glucose is exerted at the transcriptional level [12], changes in mRNA and protein stability are also involved in the process [13,14].

Ammonium assimilation in yeast occurs through its incorporation into glutamate, the source of nearly 80% of all cellular nitrogen [15]. Growth on ammonium causes a decrease in the activities of the enzymes used to assimilate less favourable nitrogen sources. This phenomenon is termed nitrogen catabolite repression, although the effect is not as well characterised as its carbon counterpart, particularly with respect to sudden changes in ammonium availability. Much less is known of the cellular response to sudden changes in the concentration of ammonium or other nitrogen sources available to the cell. A complex regulatory scheme is invoked in diploid yeast cells when they are deprived of nitrogen, this can result in pseudohyphal growth, a process regulated by a set of transcriptional activators and repressors including Phd1p, Hms1p, Mga1p and Msn1p [16]. It should be noted that, while ammonium is not one of the most preferred nitrogen sources for *S. cerevisiae*, the yeast grows well on ammonium and its presence evokes nitrogen catabolite repression [17]. Ammonium is taken via two high-affinity permeases (Mep1p and Mep2p) as well as by a low-affinity permease (Mep3p). The expression of the *GDH1*, *GLN1*, and *GAP1 *genes is regulated by the concentration of ammonium present in the growth medium [17,18]. The expression of nitrogen-regulated genes is controlled by both positively (Gln3p and Nil1p) and negatively acting proteins (Nil2p and Dal80p). In addition, it has been shown that the TOR kinases play an essential role in preventing the expression of nitrogen-regulated genes [17], and they probably have an important integrative role.

Several investigations of the transient responses of yeast metabolism to a sudden change in nutritional availability have been carried out. Kresnowati *et al*. [3] have investigated the transient short-term transcriptome and metabolome response of yeast cells to glucose perturbation in chemostats and have indicated that both the transcriptomic and metabolomic changes mediate two kinds of response - one concerned with the transition from fully respiratory to respiro-fermentative metabolism and the other with the increase in growth rate that is the consequence of an increase in nutrient supply. Ronen and Botstein [4] have investigated the transient transcriptional response to switching carbon sources between galactose and glucose and concluded that experimental designs that involve short-term transient perturbations may be useful in understanding dynamic metabolic regulatory networks. The transient response to nitrogen catabolite repression was investigated by introducing an ammonium pulse into a glutamine-limited culture [18] and showed that the ammonium-induced repression was not due to a generalised stress response but, instead, represented a specific signal for nitrogen catabolite regulation. The effect of sulphate or phosphate limitation in the growth medium, together with uracil and leucine deficiency, was also investigated and it has been deduced that the cells adjust their growth rate to nutrient availability and maintain homeostasis in the same way in both batch and steady-state conditions [19].

In this study, the dynamic re-organization of yeast's cellular activity was analyzed by following the short- and long-term transcriptomic response to a sudden relaxation of either carbon and nitrogen limitation by an impulse of glucose or ammonium, respectively. The experimental design was such that the specific perturbation was uniquely introduced into an otherwise carefully controlled environment. Thus a glucose impulse was given to a steady-state glucose-limited culture and an ammonium impulse to a corresponding ammonium-limited steady-state culture. The response of the yeast cells was monitored at the transcriptomic level until the steady state was re-established. Thus the time-scale of this investigation ranged from seconds to hours, allowing the elucidation of both the metabolic and regulatory switches that enable yeast cells to adapt to, and recover from, a transient change in nutrient availability. We believe that this study makes a significant contribution to our understanding of nutritional control in yeast since the response is studied over both short and long time-scales for two different nutrients under well-controlled physiological conditions.

## Results and discussion

The immediate, as well as the adaptive (long-term), response to the release from nutritional limitation, followed by the system's slow return to the nutrient-limited steady state was investigated using a systems biology approach. Glucose (as a carbon source) and ammonium (as a nitrogen source) were injected into their respective nutrient-limited cultures in two matched fermenters operated in fully controlled chemostat mode. Samples for transcriptome analysis were taken at different time intervals, ranging from seconds to hours, until the culture had reached a second steady state.

### Correlation analyses of genome-wide expression profiles

**Figure 1:**
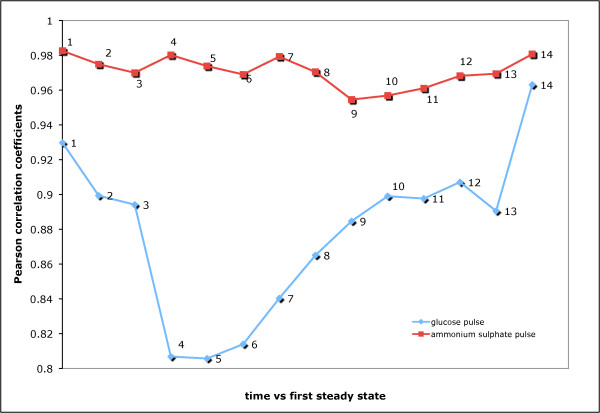
**Correlation analysis of genome-wide transcriptional response**. Each data point corresponds, on the x-axis, to samples collected at 20 sec, 40 sec, 60 sec, 8 min, 16 min, 24 min, 32 min, 1 hr, 2 hr, 3 hr, 4 hr, 5 hr, 7 hr, 2^nd ^steady state after release from the nutrient limitation represented by the 1^st ^steady state. The y-axis corresponds to the measure of correlation between the specific time point indicated in the x-axis and the 1^st ^steady state using Pearson correlation as the distance metric.

The change in the transcriptional programme of *S. cerevisiae *upon suddenly switching to a surplus of a single, previously limiting, nutrient was first investigated by comparing the array data to the preceding glucose- or ammonium-limited steady state using Pearson correlation coefficients. Introduction of glucose into the limiting medium was observed to have a pronounced and immediate effect, with a continuous decrease in correlation until the 16^th ^minute after the injection, transcript levels determined in later samples were found to be more correlated with those observed at the first steady state (Figure 1). In contrast, the transcriptional response of the ammonium-limited cells to an ammonium impulse was more subtle, with the Pearson correlation coefficient between each sample and that from the preceding steady state always >0.95 (Figure 1). It had been reported previously that carbon limitation evoked a more profound transcriptional response from yeast than other limitation for other primary nutrients, i.e. nitrogen, sulphur or phosphorus [20,21].

The gravimetrically determined biomass values and the optical densities at two steady states that were approximately 100 hr apart indicated that a significant change (loss or gain) of fitness was not detected (p-value > 0.01). The correlation between the expression profiles of two steady states was determined to be the highest among the individual profiles at each time point and the first steady state. There are *ca*. 50 generations between collecting the initial sample in the first steady state and the final sample in the second steady state. Thus, these observations indicate that spontaneous mutations during the course of fermentation are not a significant confounding factor population and so justify a detailed investigation of the transient response of the transcriptome.

### Temporal organization of the global transcriptional response

**Figure 2:**
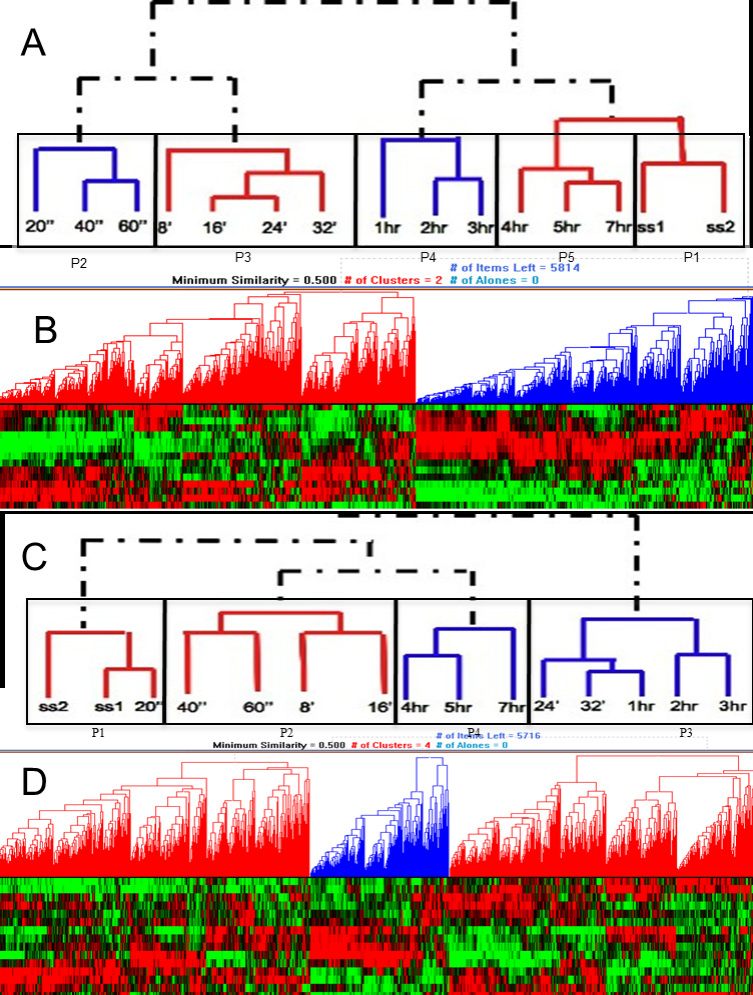
**Hierarchical clustering of the dynamics of liberation from glucose limitation (A, B) and from ammonia limitation (C, D)**. The clustering of the time points (A) and the genes (B) for carbon catabolite repression and the clustering of the time points (C) and the genes (D) for nitrogen catabolite repression are presented from top to bottom of the Figure. The clustering of the genes resulted in two major clusters (B) in the case of carbon catabolite repression (indicated in red and blue) and three major clusters (D) in the case of nitrogen catabolite repression; a similarity distance of 0.5 was used as the threshold. with the selected distance metric, as the Pearson correlation coefficient. The individual time points in the dynamic scale arranged into clusters forming distinct phases in which the transcriptome response was observed to be similar.

Correlation analysis of the transcriptome data from cells released from glucose limitation groups samples taken within the first hour following the glucose impulse and separates them from the samples from the later time points. More detailed analysis allows a further partitioning of these two main temporal clusters. The response observed in the first minute, the first hour, the first three hours, and the rest of the sampling times following the glucose impulse were found to be clustered into distinct groups, the last of which had very similar transcriptome profile to that of the preceding glucose-limited steady state. This clustering analysis revealed that the transcriptional responses obtained in the first minute were quite similar as was the case for the response in the first hour. Following the first hour, the transcriptional response was observed to be moving towards that of the steady states (Figure 2A).

The release from ammonium limitation, by providing an ammonium impulse, revealed a very different transcriptional response to that observed upon release from glucose limitation, in that the re-programming of gene expression started later and took longer both to complete and to return to the steady-state profile. Thus, the transcriptome profile recorded 20 sec. after the ammonium impulse was not significantly different to that of the preceding steady state. The profiles of cells collected 40 and 60 sec post-impulse were clustered with those from the 8 and 16 min samples and were still closely related to the steady state. The main impact of the ammonium impulse on gene transcription is seen in the period between 24 min. and 3 h. post-impulse, while the period 4-7 h. post-impulse represents a slow return to the steady-state profile (Figure 2B).

**Figure 3:**
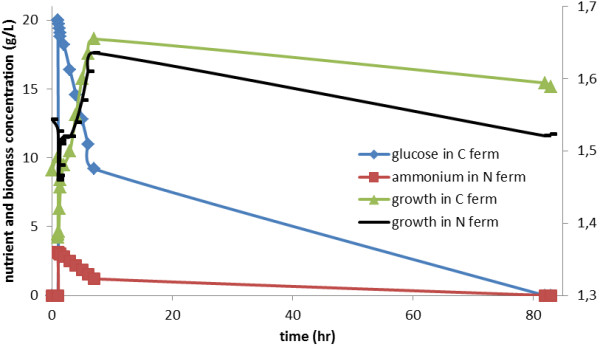
**Dilution of pulse**. Changes in the concentration of the limiting nutrient supplemented by an impulse in the chemostat as modelled by an ordinary differential equation. The cellular uptake of the nutrient for cellular growth and maintenance requirements was excluded from the model. The actual dynamics of the biomass concentration within the growth vessel is also provided. Two different scales are used on the y-axis for biomass and nutrient concentrations.

The individual temporal transcriptional profiles were also clustered via self-organizing maps to distinguish the general dynamic trends in transcriptional response of yeast cells, growing in either glucose- or ammonium-limiting chemostats at steady state, to a glucose or ammonium impulse. The transcriptome profiles fall into 81 clusters in the response to glucose perturbation and 49 clusters in that to ammonium perturbation (taking into account confidence intervals about the centroids). The impact of the impulse can be expected to last for 7 h at a dilution rate, D = 0.1 h^-1 ^(Figure 3). This figure will be the same for both the glucose and the ammonium impulses and mid-length and longer-term responses were observed at similar times for both perturbations. However the short-term responses to the two impulses differed markedly. The short-term response to the ammonium impulse started later and was more prolonged than that to glucose. Moreover, the ammonium impulse triggered an oscillatory, rather than a sustained response in some of the transcript levels.

### Genes showing a significant change in expression level in response to the nutrient impulses

Gene ontology (The Gene Ontology Consortium, 2000) biological process terms associated with genes showing a significant change in their transcript levels in response to a nutrient impulse are shown in Table 1.

**Table 1:** Gene Ontology (GO) Annotations to Differentially Expressed Genes

Significantly Associated Process GO Term	p-value	Fraction of Differentially Expressed Subset Associated with the GO Term	Fraction of Transcriptome Associated with the GO Term
**Carbon Catabolite Repression**			

Carboxylic metabolic processes	4.07 × 10^-21^	74/372	344/6353

Aspartate family amino acid metabolic processes	1.09 × 10^-6^	17/372	48/6353

Glutamine family amino acid metabolic processes	2.35 × 10^-4^	11/372	27/6353

Methionine family amino acid metabolic processes	1.69 × 10^-3^	10/372	26/6353

Serine family amino acid metabolic processes	9.85 × 10^-3^	13/372	37/6353

Purine metabolic processes	2.37 × 10^-9^	17/372	35/6353

Glycolysis	4.20 × 10^-8^	13/372	22/6353

Oxidative phosphorylation	4.79 × 10^-8^	18/372	46/6353

Alcohol catabolic processes	1.19 × 10^-7^	19/372	54/6353

Energy coupled proton transport	3.81 × 10^-7^	11/372	17/6353

**Nitrogen Catabolite Repression**			

glycolysis	3.95 × 10^-16^	15/369	22/6353

gluconeogenesis	5.85 × 10^-6^	9/369	15/6353

Proton transport	8.02 × 10^-6^	11/369	21/6353

Oxidative phosphorylation	2.98 × 10^-4^	14/369	46/6353

Aspartate family amino acid metabolic process	5.38 × 10^-4^	14/369	48/6353

Amino acid and derivative metabolic process	4.19 × 10^-3^	36/369	273/6353

#### Response to the glucose impulse

A glucose impulse was found to elicit significant changes in the transcript levels of 372 genes which are associated with the following biological process terms: 'carboxylic acid metabolic processes'; 'aspartate, glutamine, methionine and serine family amino acid metabolic processes'; 'purine metabolic processes'; 'glycolysis'; 'oxidative phosphorylation'; 'alcohol catabolic processes'; 'energy-coupled proton transport' (Table 1) (Additional file 1). Kresnowati *et al*. [3] studied the changes in transcript levels during the first 6 min following a glucose impulse and have also reported significant changes in transcript levels belonging to energy, purine ribonucleotide, amino-acid metabolism, and signal transduction functional categories in the MIPS classification as a short-term response to shifting from glucose limitation to conditions where glucose was in excess.

**Figure 4:**
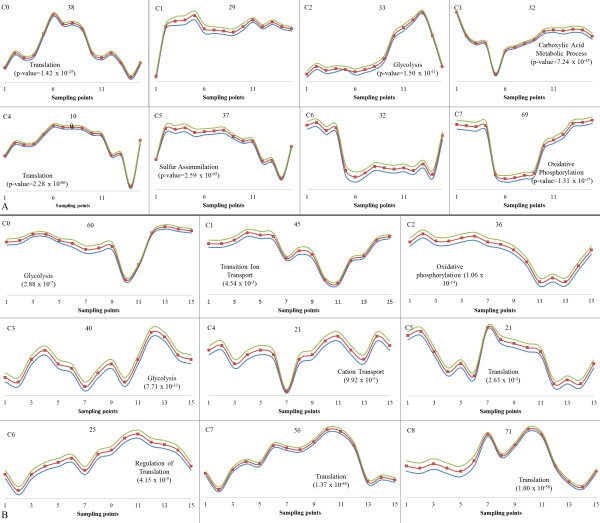
**Clustering of Significantly Expressed Transcripts in Glucose (A) or Ammonium (B) Perturbations**. The self-organization of the dynamic response of the differentially expressed transcripts around a 2 × 4 arrangement of 8 imaginary points in space in the case of carbon catabolite repression and a 3 × 3 arrangement of 9 imaginary points in space in the case of nitrogen catabolite repression are presented. The cluster number is indicated in the top left corner of each cell. The number of genes in each cluster, which is formed around the imaginary points in space with an acceptable confidence interval, is indicated in the top centre and each red square represents a time point. The blue and yellow curves represent the confidence interval around the centroids.

Transcripts that showed a significant response to the glucose impulse were placed into 8 co-responding clusters (c0 to c7; Figure 4A) using self-organizing maps [22] (Additional file 2). Six of these clusters could be associated with a biological process GO term. Glucose stimulated the expression of 138 genes significantly associated with 'translation' term and the maximum response was recorded within the first hour following the impulse (c0 and c4). For 33 genes, the increase in their transcript levels occurred later, reaching its highest level in the last three hours (c2, Figure 4A); this cluster was enriched for genes associated with the term 'glycolysis'. A group of transcripts significantly enriched with in carboxylic acid metabolic processes were immediately down-regulated with excess glucose in the fermentation medium, the expression levels slowly recovering to the initial carbon-limited state after the first 10 minutes following the glucose pulse (c3). The glucose impulse also rendered the expression of genes associated with aerobic respiration low in the first half-hour after pulse. During this period of excess glucose, the expression of oxidative phosphorylation genes, including the ATP synthesis pathway, were found to be down-regulated after the first minute following the perturbation (c7). This result is also in good agreement with the observation that gene clusters exhibiting a significant enrichment in energy and metabolism MIPS functional categories were down-regulated immediately (within 120-210 seconds) after a glucose pulse [3].

Introduction of glucose also immediately stimulated the expression of genes associated with the sulphate assimilation pathway (c5) but, as the glucose levels started to decline again, the genes associated with this pathway were down-regulated gradually after the first hour and later recovered to levels similar to that of the preceding steady state. Expression of genes for transcription factors related to sulphur metabolism were also up-regulated within 5 minutes following the introduction of glucose [3]. This is most likely to be associated with methylation, reflecting the huge demand for the post-transcriptional processing of rRNA to sustain the transiently boosted growth rate.

The transcripts in clusters c1 and c6 (Figure 4A) displayed a sustained response of either up-(c1) or down-regulation (c6) throughout the experiment after the introduction of the glucose pulse. However, analysis of the genes in these clusters failed to reveal their significant (p-value < 10^-4^) enrichment for any GO biological process category. Among the genes in c1 (the cluster displaying sustained up-regulation following the glucose impulse) were a sub-group of transcripts that were related to methylation: *SAM1 *and *SAM2*, whose products are S-adenosylmethionine synthetases. It has previously been reported that an increase in growth rate requires Sam1p, and further increases results in yet more demand for methyl donors to sustain rRNA modification, also requiring higher levels of Sam2p, a close homolog of Sam1p [20]. This supports the idea that the initial stimulation of the expression of genes concerned with sulphur metabolism is associated with the increased demand for methyl donors. Five members of the 'alcohol catabolic' process, namely *PFK1*, *PFK2*, *ENO2*, *TKL1 *and *CTS1 *were also members of the up-regulated cluster c1. Cluster 6 contains genes that displayed sustained down-regulation following the glucose impulse and included several amino-acid metabolism genes: *CIT2*, *CPA2*, *IDP2*, *ARG1 *and *CPA1 *in the glutamine family amino-acid metabolic process; *LYS20*, *LYS21*, *LYS9 *and *HOM3 *in the aspartate family amino acid metabolic process; *HOM3*, *CYS4 *and *FPR1 *in homoserine metabolic process, as well as four members of the nicotinamide nucleotide metabolic process, *PYC1*, *PYC2*, *ADH2 *and *ALD4*.

Interestingly, the transcript levels of genes for glucose transporters did not go through any major change in response to a sudden shift from glucose-limited to glucose-abundant conditions. Expression of the high-affinity glucose transporters would be expected to be fully derepressed during the preceding glucose-limited steady state. However, of the genes encoding high-affinity glucose-repressible hexose transporters, only *HXT7 *displayed a significant down-regulation in the level of its transcript immediately following the pulse. Transcript levels for the other three genes encoding high-affinity glucose transporters (*HXT2*, *HXT4*, and *HXT7*) are up-regulated from 1 h post-impulse as the glucose concentration in the growth medium starts to fall.

Published values [23] for the poly (A) tail lengths of all mRNA molecules were checked in order to identify any possible differences in mRNA degradation since no direct measurement was available. The down-regulated transcripts were not found to be significantly enriched with short poly (A) tails neither for carbon or nitrogen catabolite repression with the distribution of poly (A) tail length among up-and down-regulated transcripts appearing to be random. Since the shortest mRNA half-lives in yeast were in the range of 3 to 6 minutes [24], even the transcripts of the samples taken within the first minute are likely to be the result of an increase in transcription activity rather than an effect of mRNA degradation.

#### Response to the ammonium impulse

Relieving nitrogen limitation in the fermentation with an ammonium impulse resulted in significant changes in the transcription levels of 369 genes (Additional file 3). The members of this gene set are significantly enriched for GO bioprocess annotations associated with: 'central carbon metabolism', including 'glycolysis'; 'gluconeogenesis'; 'proton transport' and 'oxidative phosphorylation'; as well as amino acid production pathways, such as 'aspartate family amino acid metabolic process' and 'amino acid and derivative metabolic process' (Table 1).

A similar clustering of genes with a significant change in their transcript levels following the ammonium impulse, using self-organizing maps, produced 9 groups with bioprocess GO terms that can be significantly associated with each subset (Figure 4B) (Additional file 4). It was observed that the cells respond to the ammonium impulse more slowly than they do to a carbon impulse.

Down-regulation of the transcripts clustered in c0 started after the first minute displaying a sharp decrease in the expression levels after the first hour. This cluster was significantly enriched for glycolytic genes whose expression levels recovered towards the second steady state. Another cluster (c1), which was also significantly enriched with glycolytic genes, exhibited a delayed up-regulated transcriptional profile. This indicated that recovery from nitrogen limitation allowed the yeast cells also to utilize glucose better, thus resulting in down-regulation of glycolytic genes in mid-length response periods and then an up-regulation towards the cessation of the effect of the pulse as the re-establishment of high glucose concentrations resulted in the cells switching back into fermentative metabolism. Clusters significantly enriched with oxidative phosphorylation and trans-membrane ion transport processes were observed to display a down-regulation trend having the most distinct down-regulation between the 3^rd ^and the 5^th ^hours, recovering towards the second steady state (c2 and c4, respectively). This might have been due to the presence of excess glucose repressing respiration-related events during this latter period.

The transient abundance of ammonium led to an up-regulation of genes concerned with the process, and regulation, of translation. This up-regulated expression profile was displayed at the minute and hour timescales in c2, c5, c7, and c8. Clusters that were significantly enriched with 'translation process' terms (c5, c7 and c8) were also significantly enriched for 'cellular biosynthetic process' (p-value < 10^-25 ^(c2, c5), and p-value < 10^-37 ^(c8)), which indicated an up-regulation of growth-related events following a release from ammonium limitation. The induction of growth also required a higher demand for the methylation of tRNAs and rRNAs. The expression level of *SAM1 *in c2 was also observed to be up-regulated as is the case for the glucose impulse.

The expression levels of transcripts that were enriched with 'cation transport process' were sharply turned off around the first hour following the pulse (c4). Among the members of this cluster, an ammonium permease, Mep2p, works in conjunction with Pmp1p, Pmp3p and Pma1p to facilitate the trans-membrane transport of the slightly acidic ammonium during the uptake of the nitrogen source. This might have been due to the fast consumption of ammonium at that time, altering the intracellular pH, which resulted in the down-regulation of the relevant genes, only to be up-regulated again at later time points.

### Dynamic transcriptional reprogramming of the cell during the transition created by a nutrient impulse

Temporal organization of dynamic regulatory events within the transcriptional response of yeast cells to a nutrient impulse was investigated using two different systems-based approaches, namely Dynamic Regulatory Event Miner (DREM) [25] and Negative Positive Network Analysis (NP) [26]. A hidden-input/hidden-output Markov model integration of protein-DNA interactions and dynamic transcriptome data has been used in the Dynamic Regulatory Events Miner for identifying bifurcation events in the time series, where sets of genes which previously had roughly similar expression level diverge under the regulation of transcription factors that are selectively responsible for the controlling the expression of a certain subset of genes. A network-based modular approach was adapted to incorporate dynamic transcriptome data with protein-protein and protein-DNA interactions in the Negative-Positive Network Analysis in order to identify modular activity in response to catabolite repression.

#### DREM analysis

The dynamic programming of the cells in response to nitrogen and carbon catabolite repression was identified using DREM [25]. The dynamic reprogramming of the cells in response to a perturbation causing a change in nutrient availability exhibits a more complex pattern when carbon limitation is relieved than when ammonium was added to nitrogen-limited culture.

**Figure 5:**
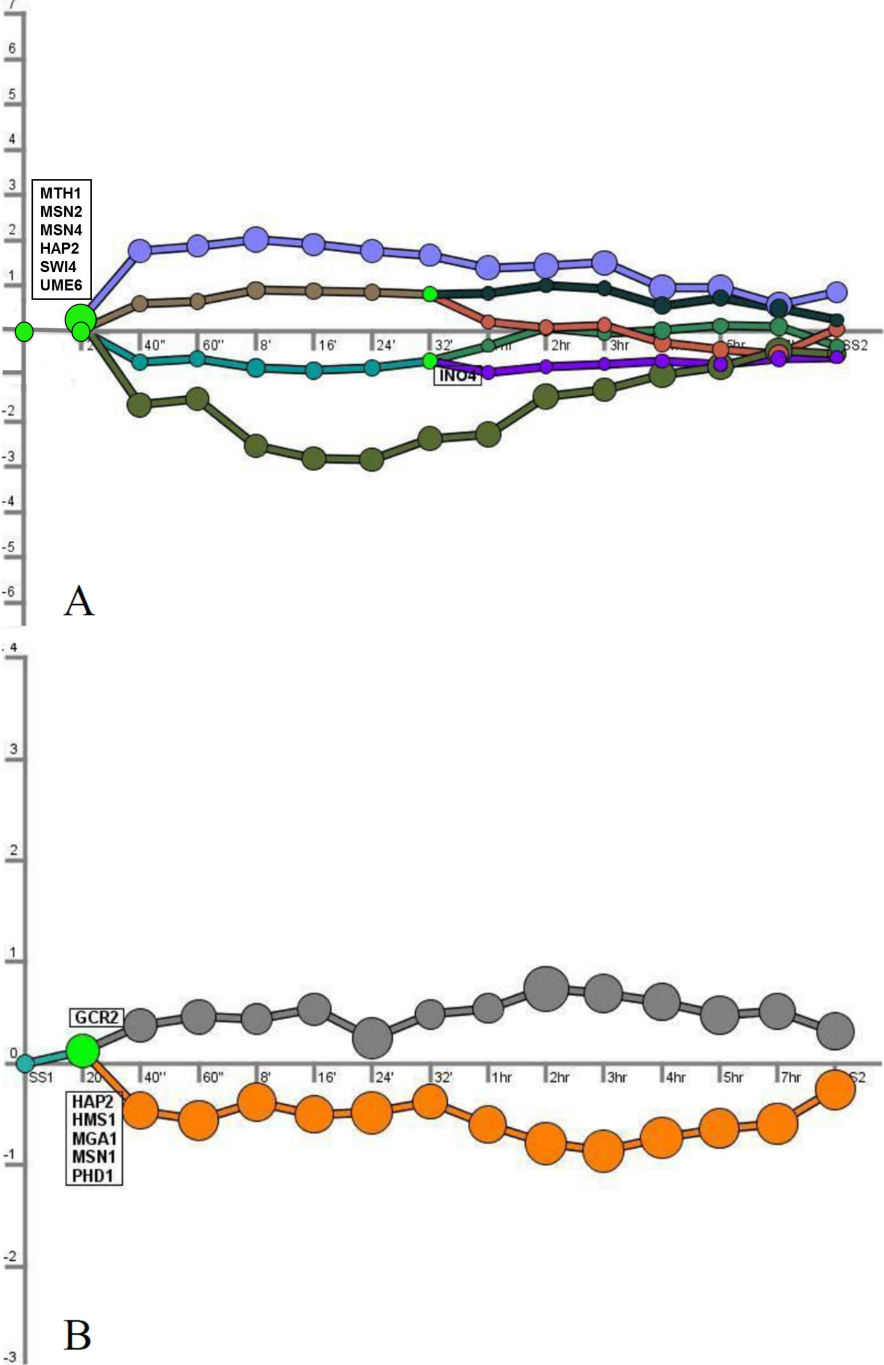
**Identification of bifurcation points in the case of carbon catabolite repression (A), or nitrogen catabolite repression (B)**. Dynamic regulatory map based on time-series gene expression data and interaction data that associates transcription factors with the genes they regulate, highlighting bifurcation events in the time series. Transcription factors selectively responsible for the regulation a certain subset of genes causing these bifurcations are also shown in the Figure. Each time point on the x-axis corresponds to the 1^st ^steady state, 20 sec, 40 sec, 60 sec, 8 min, 16 min, 24 min, 32 min, 1 hr, 2 hr, 3 hr, 4 hr, 5 hr, 7 hr post-impulse and the 2^nd ^steady state attained following release from limitation. The major paths and splits in the time series data were constructed by the genes that are assigned to these paths through the model. Each node is associated with a Gaussian distribution determining its y-axis location on the map. The area of a node is proportional to the standard deviation from the Gaussian distribution. A relatively small node implied the expression of the genes going through that node will be tightly centered around the node. Bright green nodes represent split nodes, from which multiple paths diverge.

Disturbing the glucose-limited system with an impulse-like addition of glucose resulted in a quadruple bifurcation (Figure 5A). Regulators of glucose-sensing signal transduction (Mth1p), stress conditions (Msn2p and Msn4p), respiration (Hap2p), and early meiosis (Swi4p and Ume6p) were significantly responsible for this split. The transcripts in the upper up-regulated branch were significantly associated with microtubule-associated complex while the ones in the lower up-regulated branch were associated with tRNA modification. It has been reported previously that autophagosomes are attached to microtubules for their delivery to the vacuole and autophagocytosis is significantly stimulated during nutrient deprivation [27]. Release from glucose limitation may have thus caused a re-programming of the genes associated with these processes. A further bifurcation occurred at the 32^nd ^minute with the upper set of transcripts being significantly enriched for the ER membrane component while the expression of genes constituting the lower part were significantly enriched with proteasome complex. Autophagosome formation was also previously reported to be associated with the ER membrane [28]. The transcripts in the lower down-regulated branch were enriched for 'aerobic respiration' while those in the upper down-regulated branch were enriched for retrograde transport process. A new set of bifurcations was observed at the 32^nd ^minute following the glucose induction. Ino4p; a transcription factor required for de-repression of inositol-choline-regulated genes involved in phospholipid synthesis was responsible for this onset of a late response in the upper down-regulated branch. Following this split, the transcripts found in the upper division were significantly enriched for G1-specific transcription in the mitotic cell cycle process while the genes in the lower division were enriched for the retrograde transport process. Although an association of the ER membrane and the proteasome complex could be established, the relationship between retrograde transport and aerobic respiration as well as the G1-specific transcription in the mitotic cell cycle still remains unclear and requires further investigation (Table 2).

**Table 2:** Gene Ontology Enrichment of Transcripts Following Bifurcation

	Branch of Down-regulated Transcripts			Branch of Up-regulated Transcripts		
**Case**	**Bifurcation Controlling TFs**	**Process Gene Ontology Terms**	**p-value**	**Bifurcation Controlling TFs**	**Process Gene Ontology Terms**	**p-value**

**Carbon Catabolite Repression**	Mth1p, Msn2p, Msn4p, Hap2p, Swi4p, Ume6p	ion trans-membrane transporter activity	5.3 × 10^-5^	Mth1p, Msn2p, Msn4p, Hap2p, Swi4p, Ume6p	Endoplasmic reticulum membrane	4.7 × 10^-5^

		G1-specific transcription in mitotic cell cycle	5.0 × 10^-3^		Microtubule associated complex	2.8 × 10^-3^

		aerobic respiration	8.4 × 10^-5^		tRNA modification	1.0 × 10^-3^

		retrograde transport	8.1 × 10^-3^		Proteasome complex	1.3 × 10^-3^

**Nitrogen Catabolite Repression**	Hms1p, Mga1p, Msn1p, Phd1p, Hap2p	ion trans-membrane transport	5.9 × 10^-3^	Gcr2p	ribosome biogenesis and assembly	2.9 × 10^-3^

Pulse injection of ammonium sulphate into fermentation medium resulted in a bifurcation of transcripts into up-regulated and down-regulated branches (Figure 5B). However, the observed response was delayed for 20 seconds, similar to what has been observed by the hierarchical clustering of the 20^th ^second time point with the steady state samples. A single transcription factor, Gcr2p; glycolysis regulatory protein, was significantly responsible for the split of the up-regulated branch with transcripts significantly enriched for ribosome biogenesis and assembly. Five transcription factors four of which were related to nitrogen starvation and pseudohyphal growth directly (Hms1p, Mga1p, Msn1p and Phd1p), as well as another transcription factor activating respiratory gene expression and a member of the complex facilitating the cross-pathway regulation of carbon and nitrogen metabolisms [28]; Hap2p were significantly associated with the down-regulated branch of transcripts which were enriched for ion trans-membrane transport (Table 2).

#### NP analysis

Genes, whose expression response to an environmental perturbation was either correlated or anti-correlated, are more likely to take role in the same processes [29]. In order to identify the biological phenomena underlying the transient changes observed during carbon and nitrogen catabolite repression, NP analysis was conducted (Additional file 5). The interaction networks consisting only of the nodes, whose expression profiles are either negatively or positively correlated, for carbon (C-NP) and nitrogen (N-NP) catabolite repression indicated that although an approximately similar number of correlated edges were present in both networks, a smaller number of anti-correlated edges were observed in the case of the N-NP network (Table 3). Both networks were enriched in the number of transcription factors (TF), thus in the amount of regulatory information.

**Table 3:** Network reduction via NP-analysis

Number of	Reference network	C-NP network	N-NP network
**Nodes**	5539	3419	2016

**Edges**	59784	12286	9164

**Co-regulated edges** **(PCC>0.7)**	-	8762	8868

**Anti-regulated edges** **(PCC<-0.7)**	-	3524	296

**Figure 6:**
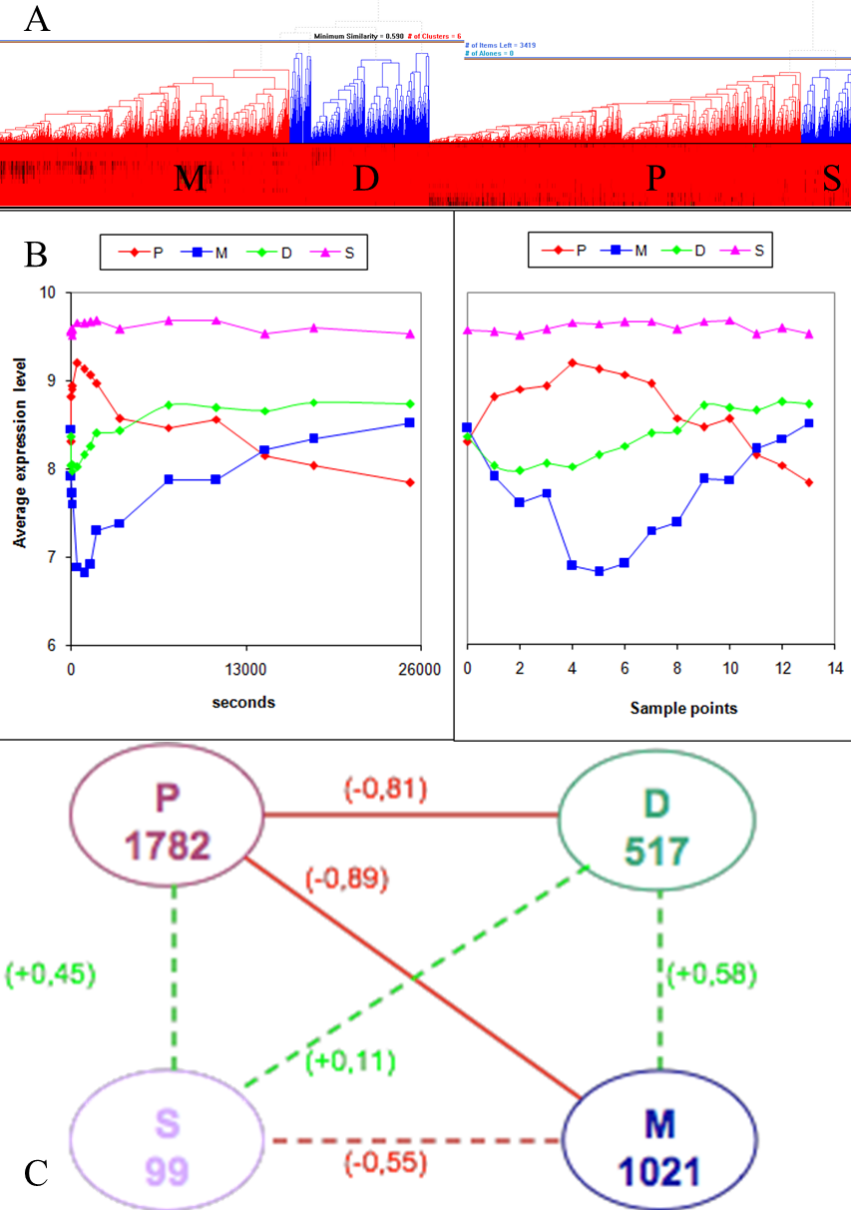
**Investigation of the C-NP network**. The modules P, M, S and D as determined by hierarchical clustering taking inter-cluster anti-correlated links into consideration (A), average dynamic expression profiles of the clusters (B), average inter-modular PCC values (C). The nodes in (C) indicate the modules with the number of genes in each module presented in the nodes. The edges indicate correlation (green dashed) and anti-correlation (red solid); the correlation coefficients are provided in parentheses.

The reduced networks were then organised into modules of correlated clusters based on the dynamic expression profiles of the genes such that less than 1% intra-cluster anti-correlated links were allowed in the clusters (modules). The C-NP network was observed to split into four modules namely; P, M, S and D. The average gene expression profiles for the clusters indicated an anti-correlation among modules P-M, P-D and S-M and a positive correlation was observed among the cluster pairs P-S and D-M, as well as among D-S (Figure 6). Due to the highly co-expressed nature of the N-NP network, only two modules were identified and the percentage of anti-correlated edges between the two modules was only 35% indicating that the genes in these two modules were operating in concert rather than in an opposite manner. Since these two clusters acted as a single global module, the N-NP network was excluded from further analysis.

The modules in the C-NP network were analysed for their enrichment in GO process terms using the detection parameter (dp). The genes in module P were enriched with growth-related GO process whereas the genes in module M were enriched in energy metabolism and redox metabolism. The smallest module, S, has more significant annotations to GO terms related to cellular proliferation and module D was related to transport and trafficking in the cell (Table 4). The indirect associations that would be determined by GO_slim terminology allowed the identification of the unknown biological process GO_slim term in module M with a cluster frequency of 19.9%. Growth was induced by the glucose impulse and a noticeable set of unknown-genes was reported to be down-regulated under this condition. Similar case reports of down-regulation of unknown genes in increasing growth conditions [20,30] indicate that these unknown genes require further investigation in terms of fitness phenotype.

**Table 4:** GO biological process terms of modules with top 10 highest detection parameters

GO terms	dp
**P module**	

ribosome biogenesis	0.0650

Transcription	0.0231

regulation of transcription, DNA-dependent	0.0172

Transport	0.0135

rRNA metabolic process	0.0135

regulation of translation	0.0125

Translation	0.0110

rRNA processing	0.0077

Cell cycle	0.0038

**M module**	

transport	0.0292

protein transport	0.0086

cellular response to heat	0.0079

oxidation reduction	0.0048

regulation of transcription, DNA-dependent	0.0032

ubiquitin-dependent protein catabolic process	0.0031

Transcription	0.0029

Translation	0.0026

**S module**	

Translation	0.1253

Transport	0.0745

regulation of translation	0.0369

regulation of transcription, DNA-dependent	0.0331

Transcription	0.0262

protein transport	0.0199

Cell cycle	0.0172

DNA repair	0.0146

Glycolysis	0.0123

chromatin modification	0.0102

**D module**	

Transport	0.0548

regulation of transcription, DNA-dependent	0.0259

protein transport	0.0182

Transcription	0.0169

ubiquitin-dependent protein catabolic process	0.0102

protein amino acid phosphorylation	0.0098

regulation of translation	0.0094

vesicle-mediated transport	0.0083

Translation	0.0055

The transcription factors among the module interface genes, which had inter-modular interactions with genes from other modules, were investigated in terms of their significance in inter-modular communication. 28 out of 32 transcription factors in module P were interface genes that were interacting with module D, indicating the importance of inter-modular regulation of cellular growth and cellular trafficking. Growth and energy metabolism, on the other hand were observed not to possess enriched regulatory communication. Rather, the regulation of cellular trafficking enabled the indirect communication between these two processes. Only two TFs were identified in Module S, which was associated with cellular proliferation and none were identified as interface genes.

## Conclusions

We have examined, over both short and long time-scales, the dynamic re-organization of gene expression in *S. cerevisiae *cells in response to a sudden relaxation of either carbon or nitrogen limitation using a system-based integrative approach. The observation of the genome-wide response at both levels, in a wide-ranging time span from seconds to hours, revealed metabolic and regulatory switches of yeast cells to adapt to and recover from an impulse-like perturbation.

The transcriptional response to impulse like addition of glucose was immediate whereas the response to ammonium was delayed for approximately 20 seconds. A larger change in the magnitude of expression of differentially transcribed genes was observed in response to carbon catabolite repression than to nitrogen catabolite repression. The transcriptional response was time-scale dependent. The response to glucose perturbation was different in the first minute, the first hour, the early hours and the late hours post-impulse both from the glucose-limited steady-state expression levels and from each other. On the other hand, the 20 seconds delay in response to ammonium perturbation caused a shift in the timescale with the remaining seconds-scale samples responding in a similar fashion to those from the early minutes, and the remaining minutes-scale samples responding in a similar fashion to those from the early hours.

The temporal distribution of priority of the biological processes was observed from the expression levels of the genes that were significantly associated with these processes. The most immediate response to the impulse-like addition of glucose was observed as the immediate down-regulation of the respiration-related transcripts within the first minute of induction. The up-regulation of growth-related transcripts that were significantly associated with translation and sulphate assimilation processes followed this response, reaching a maximum level of expression within the first half hour post-impulse, indicating that the increased demand for methyl donors, which are required for translational machinery, was met almost simultaneously with translation. The up-regulation of the glycolytic genes was the most delayed response. The expression of only one high-affinity hexose transporter, *HXT7*, was decreased in response to a surplus of glucose, possibly indicating a preferential use of this particular gene as a response to sudden changes in the availability of glucose. *HXT7 *was previously reported as having the highest affinity for glucose [31] and being the high affinity transporter, whose repression was not mediated through Mig1p [32]. The selective down-regulation of this gene might be an indicator of a Mig1p-independent transient response to changes in the extracellular availability of glucose. Interestingly, the expression levels of the genes involved with central carbon metabolism were significantly affected by the impulse-like addition of ammonium. This might have resulted from the cross-pathway regulation of carbon and nitrogen metabolisms in yeast through common transcription factors including the HAP complex. In fact, dynamic regulatory analysis identified Hap2p as the sole common transcriptional regulator of the response to either one of the two different nutritional perturbations. The down-regulation of the expression of glycolytic genes could indicate that the yeast might actually perceive the relief from ammonium limitation as a release from an environment of excess glucose, possibly explaining this combinatorial change as a relief actually from carbon catabolite repression in addition to being subjected to nitrogen catabolite repression.

Dynamic regulatory analysis was able to reveal divergence patterns in the temporal profiles of gene expression on the basis of cellular component and localization criteria, specifically highlighting different patterns for microtubule-associated complex, endoplasmic reticulum and the proteasome complex as a response to carbon catabolite repression. The presence of a relationship between the expression levels of the genes associated with retrograde transport and aerobic respiration as well as the G1-specific transcription in the mitotic cell cycle could be identified although the detailed mechanics and the identification of common transcription factors regulating these processes require further investigation.

Dynamic correlated network analysis revealed that yeast's response to nitrogen catabolite repression could be represented as a single global module, but as four complementary and interacting modules in charge of growth, energy, transport/trafficking, and cellular proliferation in response to carbon catabolite repression. The interactions among the modules were regulated by inter-modular transcription factors. Specifically, the regulation of cellular trafficking and transport acted as a bridge in the regulation of energy and growth metabolisms. The down-regulation in the expression of a set of unknown genes as a response to growth-inducing perturbations requires further investigation from an evolutionary perspective as these genes might be candidates in phenotypic analysis studies for the determination of haploinsufficiency or complex haploinsufficiency.

The present study revealed the importance of long-term analysis of the response to the relaxation from nutritional deprivation to understand the molecular basis of the dynamic behaviour of the cells. A further detailed systems-based study that integrates additional levels of functional genomics analyses may provide further information on the dynamic re-organization of yeast cells to changing environmental conditions.

## Methods

### Strain and Growth Conditions

Wild type BY4743 (*MAT**a***/*MATα his3Δ*/*his3Δ leu2Δ*/*leu2Δ LYS2*/*lys2Δ MET15*/*met15Δ ura3Δ*/*ura3Δ*; [33]) was cultivated in 2L fermenters (Applikon^®^) with 1L working volume under aerobic conditions in glucose-or ammonium-limited F1 media [34] in chemostat mode at a dilution rate of 0.1hr^-1^. Temperature and pH were controlled to 30°C and pH 4.5, respectively. Fermenters were stirred at 800 rpm which, together with constant air flow at a rate of 0.1 vvm, provided dissolved oxygen at ≥ 80% dO_2 _saturation at all times during cultivation.

### Pulse injections and sampling

**Figure 7:**
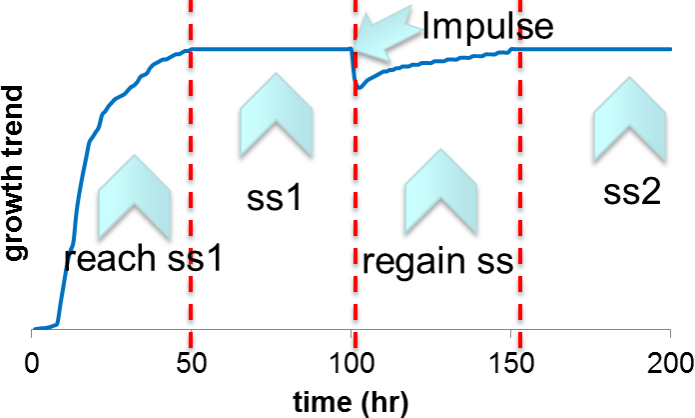
**General Scheme of Fermentation**. Following the inoculation of the fermentation medium, ca. 5 residence times elapsed before the culture reached steady state and 5 residence times were spent at steady state prior to sampling. Following the impulse, the fermentation medium is first diluted and then recovered in ca. 50 hours. The second steady sample was collected after the chemostat spent 5 residence times at steady state.

The fermentation lasted approximately 150 hours until the end of time-series sample collection following the impulse-like disturbance. After the chemostat had spent > 5 residence times at steady state, the limiting nutrient was injected into the fermentation broth aseptically to provide non-limited F1 Medium concentrations for that nutrient. 50 ml of 40% (w/v) glucose or 50 ml of 6.26% (w/v) (NH_4_)_2_SO_4 _were sufficient to provide 2% (w/v) glucose and 0.313% (w/v) (NH_4_)_2_SO_4 _concentrations in the growth media. NH_4_OH was discarded as a choice of nutrient owing to the strong base characteristic of the hydroxide ion, which would impose pH stress in the environment. It should also be noted that, since these were ammonium-limited chemostat cultures, sulphate was already in excess. Control experiments in batch culture (data not shown) have indicated that, following the ammonium impulse, phosphate (not sulphate or glucose) would become the growth-limiting nutrient. The mixing of the pulse injection was complete within milliseconds. Duplicate samples were collected at steady state prior to the impulse and as soon as the nutrient was injected, 3 samples were collected at the 20^th^, 40^th ^and 60^th ^seconds, 4 more samples were taken with 5-minute intervals within the first 20 minutes. Hourly samples were collected for five hours, and another sample was taken two hours after the last hourly sample. At that point, > 95% of the fermentation broth had been replaced with fresh medium, either by means of sampling or due to the nature of continuous cultivation. After the 210^th ^hour, when the chemostat had spent more than 5 residence times at steady state after the impulse disturbance, duplicate samples were collected at the second steady state. The general time scheme of the fermentation could be defined as follows: *ca*. 50 hours after inoculation were allowed in order to attain steady-state conditions, 50 hours was spent at steady state before sample collection, the impulse was introduced, and ca. 50 hours to regain the steady state (dynamic sampling was carried out during this period) -50 hours was allowed at the second steady state before sample collection (Figure 7).

Samples for transcriptome analyses were collected at all time points. Biomass concentration was determined gravimetrically.

### Sampling for transcriptome analysis, RNA isolation and transcriptome analysis

A culture sample (20 ml) was centrifuged at 4000 rpm for 3 min. Most of the supernatant was discarded, allowing re-suspension of cells in a small volume of growth medium. The cell suspension was released into liquid nitrogen and stored at -80°C until RNA isolation. Total RNA was isolated as described by [35]. Total RNA was qualitatively assessed on an Agilent 2100 Bioanalyser (Agilent Technologies) and quantified using Nanodrop ultra-low-volume spectrophotometer (Nanodrop Technologies). cDNA was synthesised, and double-stranded cDNA was retrieved from *ca*.15 μg of total RNA as described in the Affymetrix GeneChip^® ^*Expression Analysis Technical Manual*, using appropriate kits. cDNA was checked for quality using the Agilent 2100 Bioanalyser and was quantified using Nanodrop. Biotin-labelled cRNA was synthesized and was purified using clean up kits and it was quantified using the Nanodrop spectrophotometer before hybridization. Hybridization and loading onto Affymetrix Yeast2 arrays were carried out as described in the GeneChip^® ^*Expression Analysis Technical Manual*. The chips were then loaded into a fluidics station for washing and staining using Microarray Suite 5 with EukGe W S2v4 programme. Lastly, the chips were loaded onto the Agilent GeneArray scanner 2500 and another quality check was performed using Microarray Suite 5 [36].

### Microarray data acquisition and analysis

The raw data files were assessed with dChip software for outliers at the array level as well as at the probe-set level [37]. Different nutritional conditions (glucose and ammonium pulse experiments) were treated as different sets and were assessed for their quality control separately. RMA Express software was then used to normalize the data, again as two separate data sets [38]. The data was log_2 _transformed prior to analysis. In compliance with MIAME guidelines [39], the microarray data from this study has been submitted to ArrayExpress at the European Bioinformatics Institute under accession number [E-MTAB-643].

In order to identify transcripts whose expression significantly differed from steady-state levels following the nutrient pulse [40,41], the software package EDGE [42] was used. The 'within-class' analysis carried out by EDGE was used to look for any differential expression over time within a single group. A significance measure was then assigned to the transcript level of each gene via the Q-value methodology, using the Benjamini-Hochberg correction for the calculation of false-discovery rates. Microsoft Excel Built-In commands were used to calculate the Pearson correlation coefficients of the transcriptome at the first steady state and the rest of the sampling times. All p-values were corrected for the false-positives introduced by the multiple testing presented by many time points using Bonferroni correction and 10^-3 ^was selected as the cut-off threshold for p-values. GeneCluster 2.0 [43] was used for clustering of significant transcripts via self-organizing maps and Hierarchical Clustering Explorer (HCE) 3.0 [44] was used for hierarchical clustering purposes. The significantly enriched functional categories and the process ontology terms of the genes falling into the same cluster were determined by *Saccharomyces *Genome Database GO Term Finder tool [45] or AmiGO Term Finder tool [46]. The threshold p-value was selected as 10^-3^. Transcription factors (TF) were taken from two sources TRANSFAC Professional Gene Transcription Factor Database [47] and YEASTRACT [48] and TFs that are common in both databases were considered in further analyses.

### Dynamic Regulatory Events Miner analysis

Bifurcation points were determined using a hidden-input/hidden-output Markov model based software, DREM - The Dynamic Regulatory Events Miner as described by the authors [25]. Different nutritional perturbations, where glucose and ammonium sulphate were introduced into their corresponding limited cultures, were analyzed independently. The chromatin immunoprecipitation experiments (chip-CHIP experiments) from which the TF-gene interactions were acquired were garnered from [49,50].

### Negative-Positive Network Analysis

The Negative-Positive (NP) Network was constructed as described by [26]. The protein-protein and the protein-DNA interactome for *Saccharomyces cerevisiae *was obtained from the compilation available in the MATISSE software [51]. A total of 5539 genes for which both expression and interaction data was available were considered for analysis. The threshold for the Pearson correlation coefficient (PCC) was selected as |*PCC*| ≥ 0.7. Following network reduction, Hierarchical Clustering Explorer (HCE) 3.0 [37] was used for module identification. The largest possible modules with less than 1% intra-cluster anti-correlated links were manually dissected. MATLAB7.0 (R2006b) (The MathWorks Inc.) and Python 2.6 (Python Software Foundation) were utilized for computational purposes.

Biological meaning was assigned in terms of Gene Ontology through the use of a devised detection parameter (dp);

$$dp={\left(\frac{n}{t}\right)}^{2}$$

where *n *was the number of genes associated with the GO term and *t *was the total number of genes in the module. A GO term was used iff dp > (0.05/g) where *g *was the total number of GO terms associated with all the genes in a module [29]. GO Slim terms were also used in conjunction with dp. The definition of the interface and the core members were also adapted from [29].

The date and the party hubs were determined based on the average PCC values of the nodes. Average PCC (AvgPCC) value of a node was defined as the arithmetic average of all PCC values with its neighbors:

$$AvgPCC_{m}=\frac{\overset{n}{\underset{i}{\sum }}PCC\left(m,i\right)}{n}$$

The hubs were defined such that, if:

$$\begin{array}{l} \left|AvgPCC_{m}\right|>0.1\Rightarrow "partyhub"\\ \left|AvgPCC_{m}\right|<0.1\Rightarrow "datehub" \end{array}$$

## Authors' contributions

DD carried out the experimental study. BR carried out the transcriptome analysis. DD and MEK carried out the data analysis. DD and MEK drafted the manuscript, and DD and SGO wrote it. PP participated in the data analysis. BK and SGO participated in the design of the study and its coordination. All authors read and approved the final manuscript.

## Supplementary Material

Additional file 1 **Analysis of the transcriptome in response to glucose impulse**. The file contains data on the dynamic changes in gene expression levels across the genome, the subset of genes with a significant change in their expression, and the self-organizing maps clustering of the dynamic profiles. Click here for file

Additional file 2 **Clustering of the differentially expressed genes in response to glucose impulse**. The file contains the significantly associated process GO terms (if such exist) for the clusters of genes responsive to glucose impulse, as determined via self-organizing maps. Click here for file

Additional file 3 **Analysis of the transcriptome in response to ammonium impulse**. The file contains data on the dynamic changes in gene expression levels across the genome, the subset of genes with a significant change in their expressions, and the self-organizing maps clustering of the dynamic profiles. Click here for file

Additional file 4 **Clustering of the differentially expressed genes in response to ammonium impulse**. The file contains the significantly associated process GO terms (if such exist) for the clusters of genes responsive to ammonium impulse, as determined via self-organizing maps. Click here for file

Additional file 5 **Negative-Positive network analysis**. The file contains the reference protein-protein interaction network, the reduced networks for both the glucose and ammonium impulse cases, and the list of modules and hubs in the networks. Click here for file

## References

[B1] Shin DY Matsumoto K Iida H Uno I Ishikawa T Heat shock response of *Saccharomyces cerevisiae *mutants altered in cyclic AMP-dependent protein phosphorylation Mol Cell Biol 1987 7 1 244 250 3031463 10.1128/mcb.7.1.244-250.1987 PMC365063

[B2] Viladevall L Serrano R Ruiz A Domenech G Giraldo J Barcelo A Arino J Characterization of the Calcium-mediated response to alkaline stress in *Saccharomyces cerevisiae* J Biol Chem 2004 279 42 43614 43624 10.1074/jbc.M403606200 15299026

[B3] Kresnowati MTAP van Winden WA Almering MJH ten Pierick A Ras C Knijnenburg TA Daran-Lapujade P Pronk JT Heijnen JJ Daran JM When transcriptome meets metabolome: fast cellular responses of yeast to sudden relief of glucose limitation Mol Syst Biol 2006 2 49 16969341 10.1038/msb4100083 PMC1681515

[B4] Ronen M Botstein D Transcriptional response of steady-state yeast cultures to transient perturbations in carbon source P Natl Acad Sci USA 2006 103 2 389 394 10.1073/pnas.0509978103 PMC1326188 16381818

[B5] Causton HC Ren B Koh SS Harbison CT Kanin E Jennings EG Lee TI True HL Lander ES Young RA Remodelling of yeast genome expression in response to environmental changes Mol Biol Cell 2001 12 323 337 11179418 10.1091/mbc.12.2.323 PMC30946

[B6] Gasch AP Spellman PT Kao CM Carmel-Harel O Eisen MB Storz G Botstein D Brown PO Genomic expression programs in the response of yeast cells to environmental changes Mol Biol Cell 2000 11 4241 4257 11102521 10.1091/mbc.11.12.4241 PMC15070

[B7] Moriya H Johnston M Glucose sensing and signaling in *Saccharomyces cerevisiae *through the Rgt2 glucose sensor and casein kinase I P Natl Acad Sci 2004 101 6 1572 1577 10.1073/pnas.0305901101 PMC341776 14755054

[B8] Lafuente MJ Gancedo C Jaunlaux J-C Gancedo JM Mth1 receives the signal given by the glucose sensors Snf3 and Rgt2 in *Saccharomyces cerevisiae* Mol Microbiol 2000 35 1 161 172 10.1046/j.1365-2958.2000.01688.x 10632886

[B9] Uemra H Fraenkel DG Glucose metabolism in gcr mutants of *Saccharomyces cerevisiae* J Bacteriol 1999 181 15 4719 4723 10419980 10.1128/jb.181.15.4719-4723.1999 PMC103613

[B10] Forsburg SL Guarente L Identification and characterization of HAP4: a third component of the CCAAT-bound HAP2/HAP3 heteromer Genes Dev 1989 3 1166 1178 10.1101/gad.3.8.1166 2676721

[B11] Meijer MCM Boonstr J Verkleij AJ Verrips CT Glucose repression in *Saccharomyces cerevisiae *is related to the glucose concentration rather than the glucose flux J Biol Chem 1998 273 37 24102 24107 10.1074/jbc.273.37.24102 9727030

[B12] Gancedo JM Yeast carbon catabolite repression Microbiol Mol Biol R 1998 62 2 334 361 9618445 10.1128/mmbr.62.2.334-361.1998 PMC98918

[B13] Foat BC Houshmandi SS Olivas WM Bussemaker HJ Profiling condition specific, genome-wide regulation of mRNA stability in yeast P Natl Acad Sci 2005 102 49 17675 17680 10.1073/pnas.0503803102 PMC1295595 16317069

[B14] Kim SY Herbst A Tworkowski KA Salghetti SE Tansey WP Skp2 regulates Myc protein stability and activity Mol Cell 2003 11 1177 1188 10.1016/S1097-2765(03)00173-4 12769843

[B15] Magasnik B Ammonia Assimilation by *Saccharomyces cerevisiae* Eucaryot Cell 2003 2 5 827 829 10.1128/EC.2.5.827-829.2003 PMC219370 14555464

[B16] Lorenz MC Heitman J Regulators of pseudohyphal differentiation in *Saccharomyces cerevisiae *identified through multicopy suppressor analysis in ammonium permease mutant strains Genetics 1998 150 1443 1457 9832522 10.1093/genetics/150.4.1443 PMC1460428

[B17] Magasnik B Keiser CA Nitrogen regulation in *Saccharomyces cerevisiae* Gene 2002 290 1 18 10.1016/S0378-1119(02)00558-9 12062797

[B18] ter Schure EG Sillje HHW Vermeulen EE Kalhorn JW Verkleij AJ Boonstra J Verrips CT Repression of nitrogen catabolic genes by ammonia and glutamine in nitrogen-limited continuous cultures of *Saccharomyces cerevisiae* Microbiology 1998 144 1451 1462 10.1099/00221287-144-5-1451 9611819

[B19] Saldanha AJ Brauer MJ Botstein D Nutritional homeostasis in batch and steady-state culture of yeast Mol Biol Cell 2004 15 9 4089 4104 10.1091/mbc.E04-04-0306 15240820 PMC515343

[B20] Castrillo JI Zeef LA Hoyle DC Zhang N Hayes A Gardner DJC Cornell MJ Petty J Hakes L Wardleworth L Rhash B Brown M Dunn WB Broadhurst D O'Donoghue K Hester SS Dunkley TPJ Hart SR Swainston N Li P Gaskell SJ Paton NW Lilley KS Kell DB Oliver SG Growth control of the eukaryote cell: a systems biology study in yeast Journal of Biology 2007 6 4 10.1186/jbiol54 17439666 PMC2373899

[B21] Gutteridge A Pir P Castrillo JI Charles PD Lilley KS Oliver SG Nutrient control of eukaryote cell growth: a systems biology study in yeast BMC Biol 2010 8 68 10.1186/1741-7007-8-68 20497545 PMC2895586

[B22] Tamayo P Slonim D Mesirov J Zhu Q Dmitrovsky E Lander ES Golub TR Interpreting gene expression with self-organizing maps: Methods and application to hematopoeitic differentiation P Natl Acad Sci 1999 96 2907 2912 10.1073/pnas.96.6.2907 PMC15868 10077610

[B23] Beilharz TH Preiss T Widespread use of poly-A tail length control to accentuate expression of the yeast transcriptome RNA 2007 13 982 997 10.1261/rna.569407 17586758 PMC1894919

[B24] Carlini DB Context-dependent codon bias and mRNA longevity in the yeast transcriptome Math Biosci Eng 2005 22 6 1403 1411 10.1093/molbev/msi135 15772378

[B25] Ernst J Vainas O Harbison CT Simon I Bar-Josef Z Reconstructing Dynamic Regulatory Maps Mol Syst Biol 2007 3 74 17224918 10.1038/msb4100115 PMC1800355

[B26] Xia K Dong D Xue H Zhu S Wang J Zhang Q Hou L Chen H Tao R Huang Z Fu Z Chen YG Han JD Identification of the proliferation/differentiation switch in the cellular network of multicellular organisms PLoS Comput Biol 2006 2 e145 10.1371/journal.pcbi.0020145 17166053 PMC1664705

[B27] Lang T Schaeffeler E Bernreuther D Bredschneider M Wolf DH Thumm M Aut2p and Aut7p, two novel microtubule-associated proteins are essential for delivery for autophagic vesicles to the vacuole EMBO J 1998 17 3597 3607 10.1093/emboj/17.13.3597 9649430 PMC1170696

[B28] Dang VD Bohn C Bolotin-Fukuhara M Daignan-Fornier B The CCAAT box-binding factor stimulates ammonium assimilation in *Saccharomyces cerevisiae*, defining a new cross-pathway regulation between nitrogen and carbon metabolisms J Biol 1996 178 7 1842 1849 10.1128/jb.178.7.1842-1849.1996 PMC177877 8606156

[B29] Ohashi Y Munro S Membrane delivery to the yeast autophagosome from the Golgi-endosomal system Mol Biol Cell 2011 15; 21 22 3998 4008 20861302 10.1091/mbc.E10-05-0457 PMC2982105

[B30] Fazio A Jewett MC Daran-Lapujade P Mustacchi R Usaite R Pronk JT Workman CT Nielsen J Transcription factor control of growth rate dependent genes in *Saccharomyces cerevisiae*: A three factor design BMC Genomics 2008 9 341 10.1186/1471-2164-9-341 18638364 PMC2500033

[B31] Kasahara T Kasahara M Identification of a key residue determining substrate affinity in the yeast glucose transporter Hxt7 - A two dimensional comprehensive study J Biol Chem 2010 285 26263 26268 10.1074/jbc.M110.149716 20525688 PMC2924042

[B32] Geladé R de Velde SV Van Dijck P Thevelein JM Multi-level response of the yeast genome to glucose Genome Biol 2003 4 11 233.33 10.1186/gb-2003-4-11-233 PMC329105 14611650

[B33] Brachmann CB Davies A Cost GJ Caputo E Li J Hieter P Boeke JD Designer deletion strains derived from *Saccharomyces cerevisiae *S288C: a useful set of strains and plasmids for PCR-mediated gene disruption and other applications Yeast 1998 14 115 32 10.1002/(SICI)1097-0061(19980130)14:2<115::AID-YEA204>3.0.CO;2-2 9483801

[B34] Baganz F Hayes A Marren D Gardner DCJ Oliver SG Suitability of replacement markers for functional analysis studies in *Saccharomyces cerevisiae* Yeast 1997 13 1563 1573 10.1002/(SICI)1097-0061(199712)13:16<1563::AID-YEA240>3.0.CO;2-6 9509575

[B35] Hayes A Zhang N Wu J Butler PR Hauser NC Hoheisel JD Lim FL Sharrocks AD Oliver SG Hybridization array technology coupled with chemostat culture: Tools to interrogate gene expression in *Saccharomyces cerevisiae* Methods 2002 26 281 290 10.1016/S1046-2023(02)00032-4 12054884

[B36] Wishart JA Hayes A Wardleworth L Zhang N Oliver SG Doxycycline, the drug used to control the tet-regulatable promoter system, has no effect on global gene expression in *Saccharomyces cerevisiae* Yeast 2005 22 565 569 10.1002/yea.1225 15942933

[B37] Li C Wong WH Model-based analysis of oligonucleotide arrays: Expression index computation and outlier detection Proc Natl Acad Sci 2001 98 31 36 10.1073/pnas.011404098 11134512 PMC14539

[B38] Bolstad BM Irizarry RA Astrand M Speed TP A comparison of normalization methods for high density oligonucleotide array data based on bias and variance Bioinformatics 2003 19 2 185 193 10.1093/bioinformatics/19.2.185 12538238

[B39] Brazma A Hingamp P Quackenbush J Sherlock G Spellman P Stoeckert C Aach J Ansorge W Ball CA Causton HC Gaasterland T Glenisson P Holstege FPC Kim IF Markowitz V Matese JC Parkinson H Robinson A Sarkans U Schulze-Kremer S Stewart J Taylor R Vilo J Vingron M Minimum information about a microarray experiment (MIAME) - toward standards for microarray data Nat Genet 2001 29 365 371 10.1038/ng1201-365 11726920

[B40] Storey JD Xiao W Leek JT Tompkins RG Davis RW Significance analysis of time course microarray experiments P Natl Acad Sci 2005 102 12837 12842 10.1073/pnas.0504609102 PMC1201697 16141318

[B41] Storey JD Dai JY Leek JT The optimal discovery procedure for large-scale significance testing, with applications to comparative microarray experiments Biostatistics 2007 8 414 432 16928955 10.1093/biostatistics/kxl019

[B42] Leek JT Monsen EC Dabney AR Storey JD EDGE: Extraction and analysis of differential gene expression Bioinformatics 2006 22 507 508 10.1093/bioinformatics/btk005 16357033

[B43] Reich M Ohm K Tamayo P Angelo M Mesirov JP GeneCluster 2.0: An advanced toolset for bioarray analysis Bioinformatics 2004 20 11 1797 1798 10.1093/bioinformatics/bth138 14988123

[B44] Seo J Bakay M Chen YW Hilmer S Shneiderman B Hoffman EP Interactively optimizing signal-to-noise ratios in expression profiling: project-specific algorithm selection and detection p-value weighting in Affymetrix microarrays Bioinformatics 2004 20 2534 2544 10.1093/bioinformatics/bth280 15117752

[B45] Saccharomyces Genome Database GO Term Finder http://db.yeastgenome.org/cgi-bin/GO/goTermFinder.pl

[B46] Carbon S Ireland A Mungall CJ Shu S Marshall B Lewis S The AmiGO Hub; the Web Presence Working Group. AmiGO: online access to ontology and annotation data Bioinformatics 2008 10.1093/bioinformatics/btn615 PMC2639003 19033274

[B47] Matys V Fricke1 E Geffers R Goßling E Haubrock M Hehl R Hornischer K Karas D Kel1 AE Kel-Margoulis OV Kloos DU Land S Lewicki-Potapov B Michael H Munch R Reuter I Rotert S Saxel H Scheer M Thiele S Wingender E TRANSFAC: transcriptional regulation, from patterns to profiles Nucleic Acids Res 2003 1:31 1 374 378 10.1093/nar/gkg108 PMC165555 12520026

[B48] Teixeira MC Monteiro P Jain P Tenreiro S Fernandes AR Mira NP Alenquer M Freitas AT Oliveira AL Sá-Correia I The YEASTRACT database: a tool for the analysis of transcription regulatory associations in *Saccharomyces cerevisiae* Nucleic Acids Res 2006 34 446 451 10.1093/nar/gkj013 PMC1347376 16381908

[B49] Harbison CT Gordon DB Lee TI Rinaldi NJ Macisaac KD Danford TW Hanett NM Tagne JB Reynolds DB Yoo J Jennings EG Zeitlinger J Pokholok DK Kellis M Rolfe PA Takgusakawa KT Lander ES Gifford DK Fraenkel E Young RA Transcriptional regulatory code of a eukaryotic genome Nature 2004 431 99 104 10.1038/nature02800 15343339 PMC3006441

[B50] MacIsaak KD Wang T Gordon DB Gifford DK Stormo GD Fraenkel E An improved map of conserved regulatory sites for *Saccharomyces cerevisiae* BMC Bioinformatics 2006 7 113 10.1186/1471-2105-7-113 16522208 PMC1435934

[B51] Ulitsky I Shamir R Identification of functional modules using network topology and high-throughput data BMC Syst Biol 2007 1 8 10.1186/1752-0509-1-8 17408515 PMC1839897

